# Standardized pathology report for HER2 testing in compliance with 2023 ASCO/CAP updates and 2023 ESMO consensus statements on HER2-low breast cancer

**DOI:** 10.1007/s00428-023-03656-w

**Published:** 2023-09-28

**Authors:** Mariia Ivanova, Francesca Maria Porta, Marianna D’Ercole, Carlo Pescia, Elham Sajjadi, Giulia Cursano, Elisa De Camilli, Oriana Pala, Giovanni Mazzarol, Konstantinos Venetis, Elena Guerini-Rocco, Giuseppe Curigliano, Giuseppe Viale, Nicola Fusco

**Affiliations:** 1 Division of Pathology, IEO European Institute of Oncology IRCCS, 20141 Milan, Italy; 2 Department of Oncology and Hemato-Oncology, University of Milan, 20122 Milan, Italy; 3 Division of New Drugs and Early Drug Development for Innovative Therapies, IEO, European Institute of Oncology IRCCS, 20141 Milan, Italy

**Keywords:** HER2, HER2-low, Breast cancer, Pathology report, Biomarkers

## Abstract

Since the release of the DESTINY-Breast04 (DB-04) trial findings in June 2022, the field of pathology has seen a renaissance of HER2 as a predictive biomarker in breast cancer. The trial focused on patients with metastatic breast cancer who were classified as “HER2-low,” i.e., those with immunohistochemistry (IHC) HER2 1 + or 2 + and negative in situ hybridization (ISH) results. The study revealed that treating these patients with trastuzumab deruxtecan (T-DXd) instead of the oncologist’s chosen chemotherapy led to outstanding improvements in survival. This has challenged the existing binary HER2 pathological classification system, which categorized tumors as either positive (overexpression/amplification) or negative, as per the ASCO/CAP 2018 guideline reaffirmed by ASCO/CAP 2023 guideline update. Given that DB-04 excluded patients with HER2 IHC score 0 status, the results of the ongoing DB-06 trial may shed further light on the potential benefits of T-DXd therapy for these patients. Roughly half of all breast cancers are estimated to belong to the HER2-low category, which does not represent a distinct or specific subtype of cancer. Instead, it encompasses a diverse group of tumors that exhibit clinical, morphological, immunohistochemical, and molecular variations. However, HER2-low offers a distinctive biomarker status that identifies a specific therapeutic regimen (i.e., T-DXd) linked to a favorable prognosis in breast cancer. This unique association emphasizes the importance of accurately identifying these tumors. Differentiating between a HER2 IHC score 0 and score 1 + has not been clinically significant until now. To ensure accurate classification and avoid misdiagnosis, it is necessary to adopt standardized procedures, guidelines, and specialized training for pathologists in interpreting HER2 expression in the lower spectrum. Additionally, the utilization of artificial intelligence holds promise in supporting this endeavor. Here, we address the current state of the art and unresolved issues in assessing HER2-low status, with a particular emphasis on the score 0. We explore the dilemma surrounding the exclusion of HER2-zero patients from potentially beneficial therapy based on traditional HER2 testing. Additionally, we examine the clinical context, considering that DB-04 primarily involved heavily pretreated late-stage metastatic breast cancers. We also delve into emerging evidence suggesting that extrapolating HER2-low status from the original diagnosis may lead to misleading results. Finally, we provide recommendations for conducting high-quality testing and propose a standardized pathology report in compliance with 2023 ASCO/CAP updates and 2023 ESMO consensus statements on HER2-low breast cancer.

## Introduction: clinicopathological context

**Fig. 1:**
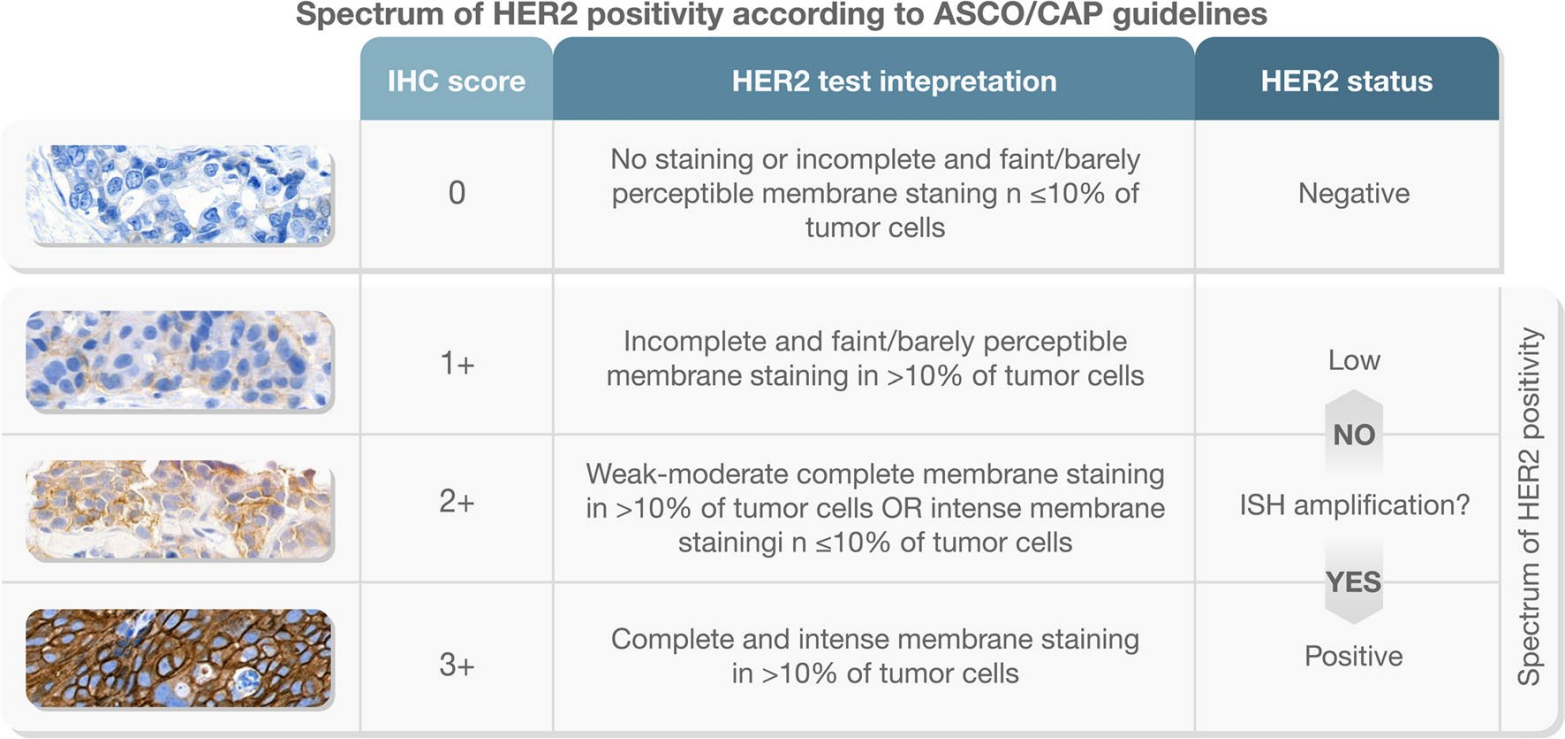
Spectrum of HER2 positivity according to ASCO/CAP guidelines. Comprehensive visual representation of HER2 expression levels in BC depicting the final HER2 status through pathological interpretation and scoring. IHC, immunohistochemistry; ISH, in situ hybridization. Breast Biomarker Reporting, CAP Cancer Protocol Templates, v v1.5.0.1 (March 2023), available at: https://documents.cap.org/documents/Breast.Bmk_1.5.0.1.REL_CAPCP.pdf

HER2 testing plays a crucial role in guiding the clinical management of patients with breast cancer (BC) [13]. The American Society of Clinical Oncology (ASCO) and the College of American Pathologists (CAP) define HER2 positivity as an immunohistochemical (IHC) score of 3 + , or a score of 2 + with gene amplification confirmed by in situ hybridization (ISH) testing [3]. Cases not meeting these criteria are considered HER2-negative [3]. This scoring system was originally developed to predict the response to early anti-HER2 therapies such as trastuzumab [9]. However, approximately 60% of hormone receptor (HR)+ /HER2− BC and 40% of triple-negative BC (TNBC) cases exhibit HER2 expression classified as score 1 + or 2 + without gene amplification (Fig. 1) [74]. Until recently, effective HER2-targeted therapies for these tumors were lacking [60]. Nonetheless, the advent of novel antibody-drug conjugates (ADCs), such as trastuzumab deruxtecan (T-DXd), which deliver cytotoxic agents to cells with low HER2 levels, has opened a new therapeutic option for this substantial BC patient population [60].

Since the approval of T-DXd for patients with advanced or metastatic HER2-low BC, the distinction between scores of 0 or 1 + has gained clinical significance, as it helps identify patients who could benefit from this treatment [53]. However, the potential challenges associated with HER2 IHC in the lower range of protein expression have not received sufficient attention [62]. It is now imperative to refine the performance of HER2 testing, considering all variables that may impact the documentation of HER2-low status in the pathology report [52]. By understanding the pitfalls of HER2 testing in light of the emerging clinical importance of HER2-low, improvements can be made in test sensitivity, specificity, and reproducibility [52, 62]. In this article, we address the current issues related to identifying HER2-low BC and offer practical recommendations for optimal testing approaches. We propose a standardized pathology report that aligns with the updated 2023 ASCO/CAP guidelines [82] and the 2023 European Society for Medical Oncology (ESMO) consensus statements [72] concerning HER2-low BC.

## Clinical and biological rationale

The clinical benefit of ADC therapy with T-DXd for patients with HER2-low BC has been established through the DESTINY-Breast04 (DB-04) trial (NCT03734029) and further supported by the DAISY trial (NCT04132960) [50]. The efficacy of T-DXd in this subset of tumors, unresponsive to trastuzumab, pertuzumab, and the first-generation ADC trastuzumab emtansine (T-DM1), is rooted in its mechanism of action [17, 35, 39]. T-DXd consists of a humanized anti-human HER2 antibody, sharing the amino acid sequence with trastuzumab, an enzymatically cleavable peptide-linker, and a proprietary topoisomerase I inhibitor (deruxtecan) [35]. Its design enables selective targeting of cancer cells expressing HER2 on the cell surface, triggering internalization of the ADC and subsequent release of the cytotoxic payload, ultimately inducing tumor cell death. The high membrane permeability of DXd also contributes to a localized bystander effect, leading to the demise of neighboring tumor cells, irrespective of their HER2 expression levels [1, 51]. HER2-low, despite not being a novel or distinct BC subtype, has been extensively studied in recent literature, consistently revealing its heterogeneity across clinical, morphological, immunohistochemical, and molecular characteristics [2, 16, 29, 33, 36, 66]. Nonetheless, HER2-low holds a distinctive biomarker status that identifies a specific therapeutic regimen (i.e., T-DXd) linked to a favorable prognosis. This unique association underscores the importance of recognizing HER2-low as a diagnostic strategy deserving of its own identity. Notably, a recent retrospective analysis of 2917 HER2-low and 2318 HER2-negative BC cases revealed a progressive increase in HER2-low tumors with rising estrogen receptor (ER) expression [71]. Conversely, ER-low tumors (characterized by 1 to 10% ER-positive cells without progesterone receptor expression) were predominantly observed among HER2-negative tumors. Given the poorer prognosis associated with ER-low tumors, these factors may introduce confounding variables when conducting prognostic analyses on HER2 low expression [27].

## Current guidelines for HER2 testing in breast cancer

According to the 2018 ASCO/CAP guideline, reaffirmed by 2023 guideline update and supported by the 2023 ESMO Expert Consensus Statements on the definition, diagnosis, and management of HER2-low BC, it is recommended to perform HER2 testing on formalin-fixed paraffin-embedded (FFPE) tissue samples derived from primary invasive BC and/or recurrent/metastatic tumors [79]. Of note, there are currently no established or widely adopted guidelines regarding the reassessment of residual tumor following neoadjuvant therapy [28, 76]. In cases of multifocal primary tumors, it is advised to perform HER2 testing on the largest lesion, with testing of smaller foci recommended if they exhibit morphological differences (e.g., distinct histology or higher grade). Recently, a 7% heterogeneity rate in HER2-positive/negative status between major and minor foci of multifocal or multicentric BC has been reported [42]; however, there is insufficient strong evidence supporting HER2 testing in all foci of multifocal disease.

The determination of HER2 status involves assessing protein expression on the tumor cell membrane using IHC or evaluating the number of HER2 gene copies through ISH techniques. IHC results should be reported based on the scoring system: score 0 (no staining or incomplete and faint/barely perceptible membrane staining in ≤ 10% of tumor cells), score 1 + (incomplete and faint/barely perceptible membrane staining in > 10% of tumor cells), score 2 + (weak/moderate complete membrane staining in > 10% of tumor cells or complete and intense membrane staining in ≤ 10% of tumor cells), and score 3 + (complete and intense membrane staining in > 10% of tumor cells). It is essential to note that a score of 3 + should be easily discernible at low-power magnification and within a homogeneous and contiguous population of > 10% invasive tumor cells. The IHC-negative HER2 group includes cases with a score of 0 or 1 + , while a score of 3 + defines HER2 positivity. In cases where IHC results are equivocal (score 2 +), further testing using ISH on the same specimen is necessary. Different ISH techniques such as fluorescence ISH (FISH), chromogenic ISH (CISH), or silver-enhanced ISH (SISH) can be employed, each with specific reporting guidelines for dual-probe and single-probe assays [58]. For dual-probe ISH assays, tumors are categorized into five ISH groups based on the HER2/chromosome enumeration probe (CEP17) ratio and the HER2 signals/neoplastic cell. The classification as positive or negative is then determined by correlating the ISH results with the IHC score. The classification for HER2 test results using dual-probe ISH involves the following ISH groups:

- ISH group 1: HER2/CEP17 ratio ≥ 2.0 and ≥ 4.0 HER2 signals/cell.
- ISH group 2: HER2/CEP17 ratio ≥ 2.0 and < 4.0 HER2 signals/cell.
- ISH group 3: HER2/CEP17 ratio < 2.0 and ≥ 6.0 HER2 signals/cell.
- ISH group 4: HER2/CEP17 ratio < 2.0 and ≥ 4.0 but < 6.0 HER2 signals/cell.
- ISH group 5: HER2/CEP17 ratio < 2.0 and < 4.0 HER2 signals/cell.

In the case of an IHC score 2 + , HER2 positivity is defined by ISH groups 1 and 3, while HER2 negativity is defined by ISH groups 2, 4, and 5. When using single-probe assays, an IHC score 2 + BC is considered HER2 positive if the average HER2 copy number is ≥ 6.0 signals/cell, and HER2 negative if the average HER2 copy number is < 4.0 signals/cell. If the results are inconclusive (average HER2 copy number ≥ 4.0 and < 6.0 signals/cell), a concurrent dual-probe ISH group 1 analysis is necessary to determine HER2 positivity.

Although the ASCO/CAP guidelines provide comprehensive details, the interpretation of the test results can be challenging [69]. This can lead to equivocal results particularly if the HER2 protein is overexpressed [19]. Similarly, ISH results may be affected by the presence of exceedingly rare chromosome 17 monosomy, which can alter the ratio of single probes and result in false positive results [11]. Additionally, a small subset of cases may exhibit significant intratumoral heterogeneity in protein expression and gene amplification, likely due to the altered biology of the HER2 oncogene/oncoprotein [48]. Importantly, no special molecular biology assays (e.g., RNA expression profiling or protein arrays) are currently recommended for HER2 testing in clinical practice (Fig. 1). Ongoing updates to the HER2 testing algorithms are available online through sources like the Breast Biomarker Reporting, CAP Cancer Protocol Templates (v1.5.0.1, March 2023 update, accessible here). Based on these guidelines, HER2-low BCs are characterized by an IHC score of 1 + or a score of 2 + with concurrent ISH groups 2, 4, or 5.

## Improving the detection and reporting of HER2-low breast cancers

HER2 is naturally expressed in normal breast epithelium, exhibiting variations in receptor quantities between cases with amplification of the HER2 gene and those without [32]. However, the capacity of IHC to detect subtle differences in receptor levels remains uncertain due to inherent limitations and technical nuances, much like other intricate IHC-based biomarkers [25, 44]. Presently, a definitive quantitative threshold to distinguish between HER2-zero and HER2-low cases is yet to be defined [20]. This not only underscores the complex challenge of precisely classifying such instances but also the need for more sophisticated methodologies in predictive pathology, including next-generation sequencing (NGS), digital droplet PCR, comparative genomic hybridization, and mass spectrometry-based assays. Despite the intricacies encountered in detecting lower levels of HER2 expression, IHC continues to be the gold standard for HER2 testing.

### Workflow in the pathology lab

**Fig. 2:**
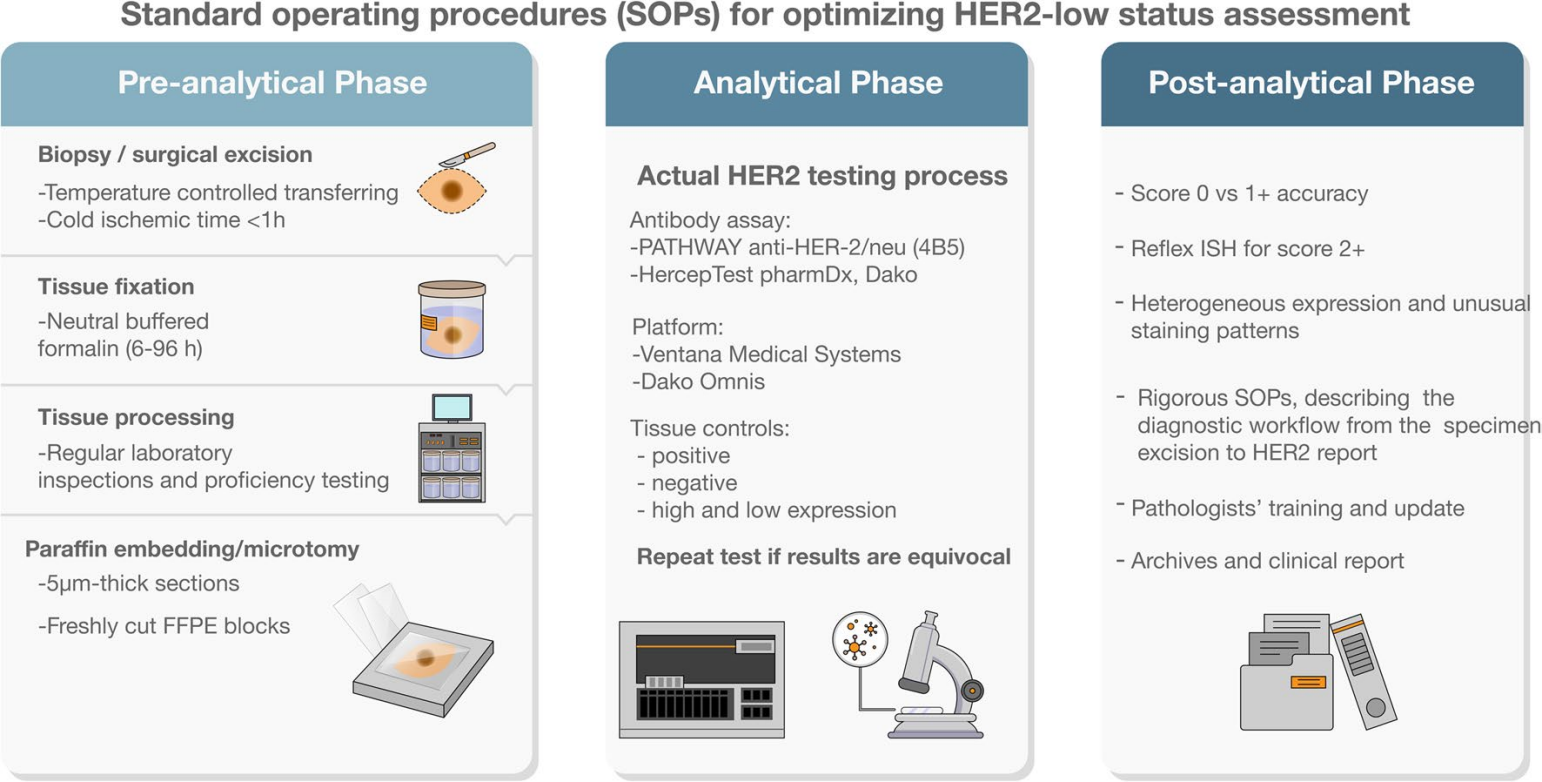
Standard operating procedures (SOPs) for optimizing HER2-low status assessment. This schematic representation provides a critical examination of the entire process for HER2 testing in pathology laboratories, encompassing pre-analytical, analytical, and post-analytical stages. Essential tips and potential pitfalls are provided for each phase, guaranteeing comprehensive guidance for pathologists and addressing critical areas throughout

Accurate and reproducible HER2 testing strategies and techniques are crucial for selecting patients who can benefit from novel HER2-ADCs [62]. To minimize the occurrence of false-negative and false-positive results, careful management of preanalytical issues is essential for both biopsy and surgical specimens (Fig. 2) [27, 86]. Various factors, including fixation, antigen retrieval, antibody clones, enzymatic activity, reaction time, temperature, and substrate concentration, can influence HER2 staining intensity and ISH sensitivity [65, 75, 85]. It is recommended to minimize cold ischemia time, which refers to the duration from tissue excision to tissue fixation, ensuring it does not exceed 1 h [78]. After macroscopic examination, the tissue should be fixed in neutral buffered formalin for at least 6 h but less than 72 h [54, 78, 83]. All testing laboratories should employ validated IHC, brightfield ISH, or FISH assays. Discrepancies in the interpretation of IHC HER2 test results can arise due to different antibody clones and antigen retrieval methods. Therefore, the current ASCO/CAP guidelines strongly recommend that laboratories performing HER2 testing participate in regular laboratory inspections and proficiency testing [81]. According to the 2018 ASCO/CAP guidelines, IHC staining should be conducted on freshly cut 2–4-µm-thick sections from representative FFPE blocks. The staining methodology, particularly antigen retrieval, and the availability of diverse antibody clones and staining platforms with varying specificity (such as PATHWAY anti-HER-2/neu (4B5), Ventana Medical Systems, S.A., Illkirch, France and HercepTest™ pharmDx, DakoCytomation, Glostrup, Denmark), can significantly impact result reproducibility and complicate the identification of HER2-low expression [67, 85]. Studies comparing the polyclonal HercepTest and the monoclonal 4B5 have shown acceptable concordance between the two methods for detecting HER2-positive BC [45, 47]. However, recent data suggests that the PATHWAY 4B5 assay may be more sensitive in diagnosing HER2-low diseases [68]. The CE-IVD-marked HercepTest™ monoclonal Ab pharmDx kit (for the Dako Omnis platform) has recently become available and demonstrated the highest overall pass rate (100%) according to the 2021 NordiQC data [57]. In a study comparing the new HercepTest (mAb) with the 4B5 assay using 119 BC samples covering the full range of HER2 IHC expression levels, the former antibody was found to be more sensitive in detecting HER2-low tumors [57]. Regardless of the assay, repeating the test in case of equivocal results may help rule out possible technical issues. Establishing and using positive and negative controls is one of the most important aspects of HER2 quality control [6, 38, 86]. Recommended controls should include low and high expression controls, which are particularly critical for HER2-low detection [37, 73]. Several factors can contribute to false-positive IHC results for HER2-low, including edge artifacts (especially in core biopsies, where cells near the tissue edges may stain more intensely than in the center), cytoplasmic positivity (which may be misinterpreted as membrane staining), and overstaining (potentially due to an inappropriate high antibody concentration) [63]. Causes of false HER2-negative results include prolonged cold ischemia time, intra-tumor heterogeneity (especially in core biopsies), and under-staining (opposite of overstaining, potentially due to excessive antibody dilution). Attention to tissue controls can help reduce both false-negative and false-positive results. Pathologists, biologists, and laboratory technicians must be well-versed in the intricate world of preanalytical issues in predictive pathology [56].

### Pathological interpretation of the test: the “score 0” challenge

With the advent of HER2-ADCs, the distinction between HER2 score 0 and score 1 + BCs has become clinically relevant, significantly impacting the routine of pathologists in biomarker testing. However, the currently employed HER2 assays were primarily designed to identify BC exhibiting HER2 overexpression, lacking specific validation for detecting HER2 low expression. A comprehensive assessment of inter-observer reproducibility for HER2-low status was conducted, encompassing a vast CAP survey involving over 1400 pathology laboratories, along with a Yale University study examining the concordance of 18 pathologists analyzing 170 BC biopsies [21]. The findings revealed a low scoring accuracy (26%) for HER2 IHC in the low expression range (score 0 vs. score 1 +), raising concerns about the potential misclassification of numerous patients for HER2-ADCs treatment in clinical practice. Notably, the level of agreement was significantly poorer compared to that observed between score 2 + and score 3 + . Due to inconsistent findings concerning the prognosis of HER2-low BC, a comprehensive retrospective cohort study was undertaken utilizing the National Cancer Database [55]. This study encompassed 1,136,016 US patients diagnosed with invasive BC between January 1, 2010, and December 31, 2019, who exhibited ERBB2-negative disease and had accessible immunohistochemistry results [55]. The investigation compared the characteristics of HER2-low and HER2-zero BC cases and revealed minimal prognostic disparities, implying that the effectiveness of HER2-directed therapies will play a crucial role in shaping outcomes, rather than inherent biological differences associated with low levels of HER2 expression. Nonetheless, encouraging outcomes surfaced from the preliminary results of a multinational, multicenter, non-interventional, retrospective study on HER2 retesting (NCT04807595) [77]. This study included 798 patients diagnosed with locally advanced or metastatic BC between 2015 and 2017, displaying canonical HER2-negative status (IHC score 0, 1 + , or 2 + without gene amplification). Following a web-based training session for pathologists involved in scoring low-end HER2 expression, who were blinded to historical HER2 scores, the original HER2 IHC-stained slides (mainly employing the Ventana 4B5 assay) were reassessed and reclassified as either HER2-low or HER2-zero. HER2 rescores were successfully obtained for 786 patients, demonstrating an 81.3% concordance between historical HER2 scores and rescores. It should be noted, however, that in the study conducted by Fernandez et al. [21], the participating pathologists were unaware that the interobserver agreement between the IHC score 0 and 1 + would be evaluated. In contrast, the observers in the NCT04807595 trial were fully aware of the investigation’s scope and, more importantly, the clinical implications of the new HER2-low category. The interpretation challenges are further complicated by the heterogeneity of HER2 expression [15, 22, 24, 43, 46]. This phenomenon is considered an independent risk factor for decreased disease-free survival and poses difficulties in selecting appropriate treatments for such patients [5, 54, 64]. In this context, the patterns of protein distribution hold significant relevance [30]. Geographical variations in HER2 staining within the same tumor, characterized by distinct patterns such as “clustered,” “mosaic,” and “scattered,” can significantly influence the identification of HER2-low BC, particularly in cases classified as HER2 “equivocal” [46, 84]. The concept of HER2 heterogeneity encompasses not only variations in HER2 expression levels but also the phenomenon of HER2 status switching and loss of HER2 expression, which can occur as a consequence of HER2-targeted therapy or following neoadjuvant treatment [23, 34, 49, 70]. In these settings, we encourage HER2 retesting [3, 10]. Recently, the change of HER2-low status from primary tumors to metastatic sites was investigated through a retrospective analysis of 554 BC [70]. Overall, HER2 discordance between primary and metastatic disease occurred in half of the cases [70]. Similarly, Miglietta et al., who assessed HER2 status in 547 patients with matched primary and recurrent samples, observed an overall HER2 discordance rate of 38%, primarily characterized by HER2-zero switching to HER2-low (15%) and HER2-low switching to HER2-zero (14%) [49]. These findings may indicate a genuine possibility that the conditions of the tissue, the test itself, or the interpretation of the test are not adequately sensitive in detecting low levels of HER2 protein expression. Considering the instability of HER2-low expression during disease progression, it is recommended to perform a biopsy of recurrent lesions if the primary tumor was previously scored as 0, whenever feasible from a clinical standpoint. Conversely, when conducting a biopsy of metastatic lesions and obtaining a score of 0, it is advisable to take into account the initial HER2 testing result of the primary tumor and reassess it if it was initially diagnosed as HER2-zero. Eligibility for T-DXd treatment is granted to patients if at least one tumor sample demonstrates HER2-low status, regardless of when the sample was obtained.

### Checklist and reporting of the results

**Fig. 3:**
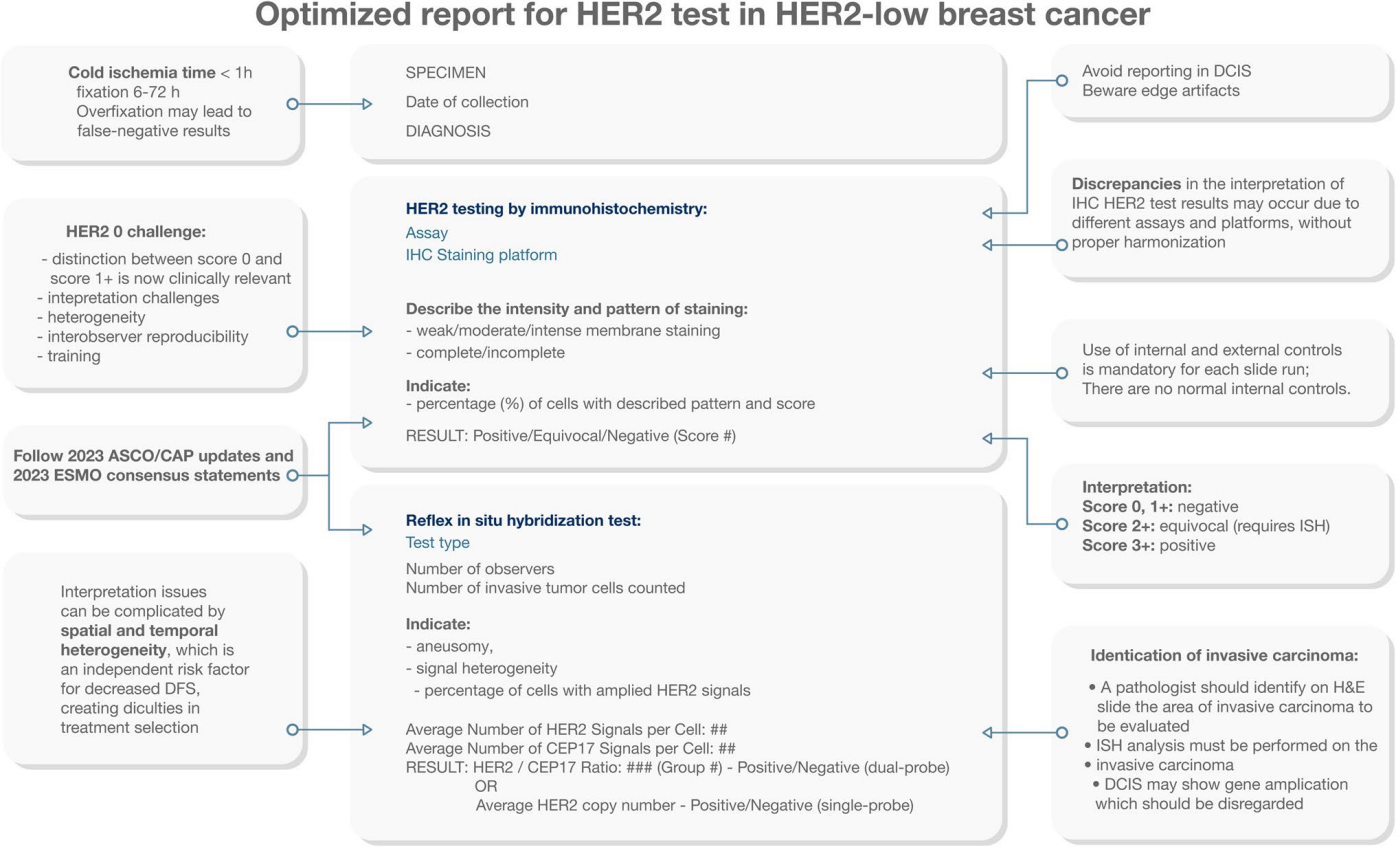
Pathology report optimization for HER2 test in HER2-low breast cancer. This refined report presents an integrated approach to accurately assess the HER2 status in breast cancer cases classified as HER2-Low in compliance with 2023 ASCO/CAP updates and 2023 ESMO consensus statements. By synergistically combining immunohistochemistry and in situ hybridization techniques, it is essential to provide a clear, precise, and comprehensive report of HER2 status to enable improved treatment decisions and personalized patient management. IHC, immunohistochemistry; CEP17, chromosome enumeration probe 17; DCIS, ductal carcinoma in situ; H&E, hematoxylin and eosin; ISH, in situ hybridization

Timely release of a clear, concise, and comprehensive pathology report plays a vital role in facilitating clinical decision-making [26, 40]. To maintain quality assurance, strict adherence to standard operating procedures (SOPs) is imperative, outlining the diagnostic workflow from specimen excision to the final HER2 report [8, 14]. Current ASCO/CAP guidelines recommend the use of Food and Drug Administration (FDA)-approved IHC/ISH assays and periodic inspections of testing laboratories [80]. Efficient organization of sample storage and laboratory documentation is crucial to facilitate retesting if necessary [7, 12, 27, 37, 41, 49]. The quality of HER2 testing results and adherence to standard operating procedures rely directly on the laboratory personnel, encompassing technicians, molecular biologists, and pathologists [4, 31, 56]. Evaluation of HER2 status should align with the current ASCO/CAP guidelines, encompassing the IHC score ranging from 0 to 3 + , intensity and pattern of staining, and the percentage of positive cells with the highest staining pattern seen in > 10% of invasive tumor cells. Moreover, although not currently recommended for routine practice outside of clinical trials, providing the percentage (10% or less) of immunostained cells in samples with an IHC score of 0 could hold future value. This approach may prove beneficial for investigating the potential advantages of new ADCs in the context of HER2-ultra low BC (defined as a score of 0 with incomplete and faint staining in  >0 and ≤ 10% of tumor cells) in forthcoming studies [61]. Methodological information, including the primary antibody used (e.g., 4B5, HercepTest GE001), should be provided, along with a statement confirming adherence to the ASCO/CAP guidelines. The ISH test should indicate the positivity or negativity of the test, specify the number of observers and invasive tumor cell counts, and offer additional details regarding aneusomy, signal heterogeneity, and the percentage of cells with amplified HER2 signals [78]. In Fig. 3, we present a proposed optimal report for HER2 testing, tailored specifically for HER2-low BC. This report aims to provide a standardized framework that promotes consistent and informative documentation of HER2 testing results.

## Conclusions and future implications

The expanding therapeutic options based on HER2 expression patterns have introduced greater complexity to HER2 testing. It is important to clarify, however, that the incorporation of the HER2-low diagnostic category should be construed as a catalyst for instigating a paradigm shift in the cognitive framework of pathologists. Rather than serving as a conduit for a radical departure from existing diagnostic modalities, this category ought to be embraced as a transformative impetus toward heightened quality of the test. It beseeches pathologists to engage in a process of meticulous scrutiny and discernment across all phases of HER2 test, particularly in scenarios where HER2 expression assumes a subdued profile, which is now clinically relevant. Indeed, the *raison d'être* of the “HER2-low classification” in the DB-04 trial was to provide a simplified reference for the inclusion criteria of BC that were enrolled in the study based on specific criteria (i.e., IHC score 1 + or 2 + /ISH negative) for treatment with T-DXd. It is important to note that HER2-low does not denote a novel BC subtype but rather serves as a descriptive diagnostic category. However, pathologists are now tasked with specifically identifying the intricate subtleties of HER2 expression dynamics across a wider continuum, an aspect that was previously deemed clinically irrelevant. Rigorous quality control and clear assessment guidelines are indispensable in this regard. Novel HER2 testing methods, such as multiplex ligation-dependent probe amplification (MLPA), show promise in overcoming methodological and biological heterogeneity [19]. Currently, the ongoing DESTINY-Breast06 (DB-06) trial (NCT04494425) is investigating T-DXd in a patient cohort with HER2 score 0, as defined by ASCO/CAP, or HER2-ultra low, including IHC values >0 and ≤ 10%. The objective is to establish the clinical validity and utility of the “HER2-zero > 0” biomarker, and to evaluate the potential clinical benefits of the drug by assessing progressively lower levels of HER2 IHC. To elaborate, the previous DB-04 trial excluded patients with an IHC score of 0, and it yielded positive results. Now, in DB-06, one-third of the patient cohort includes individuals with IHC scores ranging from more than “null” to less than 1 + . Data from this trial are expected by the end of 2023. It is likely that the trial will produce positive outcomes since it will assess the efficacy of the drug in the entire patient population, including those with scores of 0 to 1 + , 1 + , and 2 + ISH not amplified, and without any prior lines of treatment. If this scenario indeed materializes, all these complexities are likely to become futile exercises, and T-DXd to become an effective treatment option for at least two distinct groups: those with HER2 pathway activation/addiction (HER2 overexpressed or amplified) and those with HER2 protein present (HER2 not overexpressed or amplified), as most or all breast tumors contain some level of HER2 protein. However, as long as the results of the DB-06 are not made public, it is premature to change well established rules of HER2 reporting. It is crucial to develop assays that offer more quantitative measurements than IHC to examine these samples and identify potential differential benefits or responses based on quantifiable measures of HER2 protein. In this respect, artificial intelligence (AI) holds significant potential in revolutionizing HER2 testing [59]. Considering the prevalent discordance in HER2 status between primary and metastatic disease that affects a substantial portion of cases, it's prudent to consider evaluating the HER2-low status using metastatic tissues initially. This viewpoint suggests that optimizing AI models by training them on extensive HER2-stained datasets enriched with metastatic samples could enhance both precision and efficiency, thereby addressing subjectivity and variability concerns [18]. To ensure a comprehensive approach to interpreting HER2 IHC expression in the low range, ongoing education and updates remain vital components.

## Data Availability

Data sharing not applicable to this article as no datasets were generated or analyzed during the current study.
